# Supplementary material for: Antidiabetic Effects of Pediococcus acidilactici pA1c on HFD-Induced Mice
Source: Nutrients. 2022 Feb 7;14(3):692. doi: 10.3390/nu14030692 (PMC8839473; doi:10.3390/nu14030692)
Supplement: Supplementary file 1 [file nutrients-14-00692-s001.zip › nutrients-1544857-supplementary.pdf]

SUPPLEMENTARY TABLES

Table S1. Sequences of primers used in gene expression analysis.

Target gene	Forward/ Reverse	Primer sequence (5´ to 3´)	Source
GADPH	F	TCAAGAAGGTGGTGAAGCAG	(46)
	R	TCCACCACCCTGTTGCTGTA	
G6pase	F	CACCGACTACTACAGCAACAGC	(47)
	R	AGAATCCCAACCACAAGATGAC	
PEPCK	F	AGTCATCATCACCCAAGAGC	(47)
	R	GGGATGACATACATGGTGC	
GCK	F	CTTCACCTTCTCCTTCCCTGTAA	(47)
	R	AAAGTCCCCTCTCCTCTTGATAG	
IL-1β	F	TCGCTCAGGGTCACAAGAAA	(48)
	R	CATCAGAGGCAAGGAGGAAAAC	
IL-6	F	ACAAGTCGGAGGCTTAATTACACAT	(48)
	R	TTGCCATTGCACAACTCTTTTC	

GADPH: glyceraldehyde-3-phosphate dehydrogenase; GCK: glucokinase; G6pase: glucose 6 phosphatase; IL-1β: interleukine-1β; IL-6: interleukin-6; Pepck: phosphoenolpyruvate carboxykinase.

Table S2. Biochemical parameters of experimental groups

Parameter	C group	T group
FFA (nmol/µl)	0.61 ± 0.08	0.57 ± 0.08
TC (µg/µl)	3.03 ± 0.25	3.19 ± 0.49
TG (nmol/µl)	0.78 ± 0.15	0.73 ± 0.16

FFA: free fatty acids; TC: total cholesterol; TG: triglycerides.

Table S3. Correlation coefficients (Pearson's correlation analysis between study parameters

Parameters		r	*P*
FBG (log-transformed)	BW	0.2360	0.2668
	Serum GLP-1	-0.6770	**0.0003*** **
	Serum C-peptide	-0.1801	0.4226
	Serum leptin	0.4568	**0.0429***
	G6P	0.5695	**0.0037****
	PEPCK	0.6160	**0.0018****
	GCK	-0.4227	**0.0445***
Serum GLP-1	BW	-0.3409	0.1031
	FBG (log-transformed)	-0.6770	**0.0003*** **
	Serum C-peptide	-0.0011	0.9962
	Serum Leptin	-0.2516	0.2847
	G6P	-0.4504	**0.0272**
	PEPCK	-0.4801	0.0204
	GCK	-0.2910	0.1780
Serum C-peptide	BW	-0.1604	0.4757
	FBG (log-transformed)	-0.1801	0.4226
	Serum GLP-1	-0.0011	0.9962
	Serum Leptin	-0.2088	0.4058
	G6P	-0.5037	**0.0169***
	PEPCK	-0.3971	0.0746
	GCK	-0.1626	0.4812
Serum Leptin	BW	0.2476	0.2926
	FBG (log-transformed)	0.4568	**0.0429***
	Serum GLP-1	-0.2516	0.2847
	Serum C-peptide	-0.2088	0.4058
Globet cells	HOMA-IR	-0.6668	**0.0179***

Statistical significance is highlighted in bold. BW: body weight; FBG: fasting blood glucose; GCK: glucokinase; GLP-1: glucagon-like peptide-1; G6P: glucose-6-phosphatase; HOMA-IR: homeostatic model assessment for insulin resistance; PEPCK: phosphoenolpyruvatecarboxykinase;
r = Pearson's rho. * $p < 0.05$, ** $p < 0.01$, *** $p < 0.001$.
